# Reconstructing the Phylogeny of *Capsosiphon fulvescens* (Ulotrichales, Chlorophyta) from Korea Based on* rbc*L and 18S rDNA Sequences

**DOI:** 10.1155/2016/1462916

**Published:** 2016-04-17

**Authors:** Sang-Mi Sun, Seung Hwan Yang, Kirill S. Golokhvast, Bao Le, Gyuhwa Chung

**Affiliations:** ^1^Department of Biotechnology, Chonnam National University, Yeosu, Chonnam 59626, Republic of Korea; ^2^Center for Nutraceutical and Pharmaceutical Materials, Myongji University, Yongin, Gyeonggi 17058, Republic of Korea; ^3^Educational Scientific Center of Nanotechnology, Far Eastern Federal University, Vladivostok 690-950, Russia; ^4^Department of Biomedical and Electronic Engineering, Chonnam National University, Yeosu, Chonnam 59626, Republic of Korea

## Abstract

*Capsosiphon fulvescens* is a filamentous green algae in the class Ulvophyceae. It has been consumed as food with unique flavor and soft texture to treat stomach disorders and hangovers, and its economic value justifies studying its nutritional and potential therapeutic effects. In contrast to these applications, only a few taxonomic studies have been conducted on* C. fulvescens*. In particular, classification and phylogenetic relationships of the* C. fulvescens* below the order level are controversial. To determine its phylogenetic position in the class, we used* rbc*L and 18S rDNA sequences as molecular markers to construct phylogenetic trees. The amplified* rbc*L and 18S rDNA sequences from 4* C. fulvescens* isolates (Jindo, Jangheung, Wando, and Koheung, Korea) were used for phylogenetic analysis by employing three different phylogenetic methods: neighbor joining (NJ), maximum parsimony (MP), and maximum likelihood (ML). The* rbc*L phylogenetic tree showed that all taxa in the order Ulvales were clustered as a monophyletic group and resolved the phylogenetic position of* C. fulvescens* in the order Ulotrichales. The significance of our study is that the 18S rDNA phylogenetic tree shows the detailed taxonomic position of* C. fulvescens*. In our result,* C. fulvescens* is inferred as a member of Ulotrichaceae, along with* Urospora* and* Acrosiphonia*.

## 1. Introduction

*Capsosiphon fulvescens* (C. Agardh) Setchell and N. L. Gardner, filamentous chlorophycean seaweed, is found in the North Atlantic [1, 2] and in the Northern Pacific, including Korea [3] and Japan [4]. Its natural habitat is the upper intertidal regions of coastal sediments and rocky shores which it shares with the common edible seaweed* Ulva prolifera* Mueller.* Capsosiphon fulvescens*, a filamentous green algae, reproduces by biflagellated isogametes released from bisexual gametophytes [5]. This seaweed is known to be a contaminant in* Porphyra* cultivation [6]. However, in the Southwestern province of Korea, it has been consumed as food with unique flavor and soft texture to treat stomach disorders and hangovers [7], and its economic value justifies studying its nutritional and potential therapeutic effects. Several physiological studies conducted* in vitro* and* in vivo* have suggested that extracts of* C. fulvescens* had an inhibitory effect on melanogenesis in B16 cells [8], induced apoptosis in AGS gastric cancer cells [9], and reduced cholesterol levels in hypercholesterolemic rats [10]. The potential economic interest in* C. fulvescens* could justify its large-scale cultivation in both the laboratory and the field [11].

In contrast to attention to its applications, only a few taxonomical studies have been conducted regarding* C. fulvescens*. In particular, classification and phylogenetic relationships of the* C. fulvescens* below the order level are controversial. The reasons for discrepancies among classification schemes include disagreements regarding the evaluation of morphological characters. For example,* C. fulvescens* produces gametes and zoospores like those of* Ulothrix* and* Urospora* [12] while it was also considered to be closely related to* Monostroma* by Migita [5] because of discontinuous reproductive patches near the thallus apex as well as similarity of gametes. However, Chihara [4] considered that it was closely related to* Percuriaria* and* Ulva* because fronds produce zygotes that germinate directly without formation of a thick-walled zygote [4]. It is often difficult to identify symplesiomorphies characters because similar characters can be derived from convergent or parallel evolution of Ulotrichales.

Molecular systematics in seaweeds has progressed rapidly with the use of PCR coupled with sequencing methods. This molecular approach has been effective in addressing many phylogenetic questions that had not been solved using phenotypic characters. The gene for the large subunit of ribulose-bisphosphate carboxylase (*rbc*L) located in the chloroplast genome and the 18S rDNA in the nuclear genome have been extensively used for the inference of phylogenetic relationships at higher taxonomic levels because of their slow synonymous nucleotide substitution rates and strong functional constraints of* rbc*L sequence that reduced the evolutionary rate of nonsynonymous substitutions. The first report to mention the classification of* C. fulvescens* collected from North Atlantic with a molecular marker (cf. 18S rDNA) was presented by Hayden and Waaland [13]. They suggested that* C. fulvescens* was in the order Ulotrichales, which was consistent with Nagata's report [12]. In 2008, Hanic and Lindstrom [14] also used 18S rDNA sequence to prove that* C. fulvescens* and* Pseudothrix borealis* (entity formerly called* C. groenlandicus*) do not belong in the same genus* Capsosiphon*.

In this study, for the first time,* C. fulvescens* have been examined using* rbc*L gene and 18S rDNA sequences of newly collected material from different provinces in South Korea and these sequences have been employed to understand the phylogenetic position of Korean* Capsosiphon fulvescens* in the Ulvophyceae.

## 2. Materials and Methods

### 2.1. Sample Collection and Culture

*C. fulvescens* thalli were collected from five different seaweed farms located in Jindo, Koheung, Wando, and Janghuyn, South Korea, during December 2011 to February 2012. They were washed several times in clean cold seawater and kept on ice until being returned to the laboratory. Seawater samples were collected from discrete depths using 10 Niskin bottles arranged on a conductivity, temperature, and depth (CTD) rosette. The entire contents of the bottles were gravity filtered onto a 47 mm Poretics membrane filter (GE Osmonics, Fairfield, CT, USA), with a pore size of 5 *μ*m, held within a Millipore Swinnex filter holder (Millipore, Bedford, MA, USA). The filtration time varied between 30 min and 2 h; if the Niskin bottle was not completely filtered at the end of 2 h, the filter was processed, noting the amount of seawater filtered. Freshly collected plants were grown in seawater filtered medium in glass culture vessels at 8°C under 30–50 *μ*mol photons m^−2^ s^−1^, 14 : 10 h LD cycle. The species was identified microscopically, manually separated from other algae, and washed with tap water and distilled water. Authentic standard compounds were purchased from Tokyo Kasei Kogyo Co., Ltd. (Japan), and Supelco Inc. (Bellefonte, USA).

### 2.2. DNA Extraction

Whole cultured biomass of* Capsosiphon fulvescens* was freeze-dried, and genomic DNA from freeze dried materials was extracted using a modified hexadecyltrimethylammonium bromide (CTAB) method [15], in which samples (~0.5 g) were ground in 1 mL CTAB using a sterile mortar and pestle. DNA extracts were cleaned with a Wizard PCR purification system (Promega, Madison, WI, USA), according to the manufacturer's instructions.

### 2.3. PCR Amplification and Sequencing

Double-stranded amplification of the 18S rDNA and* rbc*L regions was performed in a total volume of 50 *μ*L using 1.0 *μ*L of total genomic DNA (10–20 ng) template. The PCR amplifications were performed with 1 unit of* Taq* DNA Polymerase PCR Buffer (Invitrogen), 1.5 mM MgCl_2_, 200 *μ*M dNTP mix, and 2.5 *μ*mol of each primer (PTC-200, MJ Research, Waltham, MA, USA). The* rbc*L and 18S rDNA genes were amplified using published primers and other primers designed from an alignment of available* rbc*L and 18S sequences (Table 1) with the following conditions.

The reactions were conducted using an initial denaturation at 94°C for 3 min, followed by 35 cycles each of 1 min at 94°C, 1 min at 50°C, and 1 min at 72°C, followed by final extension for 10 min at 72°C. The PCR products were analyzed by 1.2% (w/v) agarose gel electrophoresis and purified with a Wizard PCR purification system (Promega, Madison, WI, USA). The cleaned PCR products were sequenced using the ABI Prism BigDye Terminator Cycle Sequencing Ready Reaction Kit (Applied Biosystems Foster City, CA, USA) following the manufacturer's instructions. Sequences were generated in both the forward and reverse directions using an automatic sequencer ABI PRISM 310 Genetic Analyzer (Applied Biosystems Foster City, CA, USA) (see Supplementary Materials available online at http://dx.doi.org/10.1155/2016/1462916).

### 2.4. Phylogenetic Analyses

Assemblies of the newly created DNA sequences in this study were carried out using the DNASTAR program (DNASTAR, Inc., Madison, WI, USA). The coverage of sequences determined was 2x. Multiple sequence alignments were performed using the ClustalX 2.1 with default parameters and manually edited. Other sequences were obtained from GenBank using BLASTN search with the identified sequences as queries. Twenty-five* rbc*L sequences belonging to Ulvophyceae were used to construct a phylogenetic tree with 2 outgroup sequences. Thirty-three 18S rDNAs were used to construct phylogenetic tree. For phylogenetic analyses, 2 different methods were applied: neighbor joining (NJ) and maximum parsimony (MP) using software Mega v6 [18] with complete deletion of gaps. In MP analyses, nucleotide positions and character state changes were weighted equally after removing uninformative characters. ML analysis was carried out using RAxML v8 [19] with the HKY model of DNA sequence evolution and gaps were treated as unknown characters. For all 3 analysis types, branch support was assessed by bootstrapping (1000 replicates).

## 3. Results

### 3.1. Constitution of* rbc*L Phylogenetic Trees

**Figure 1:**
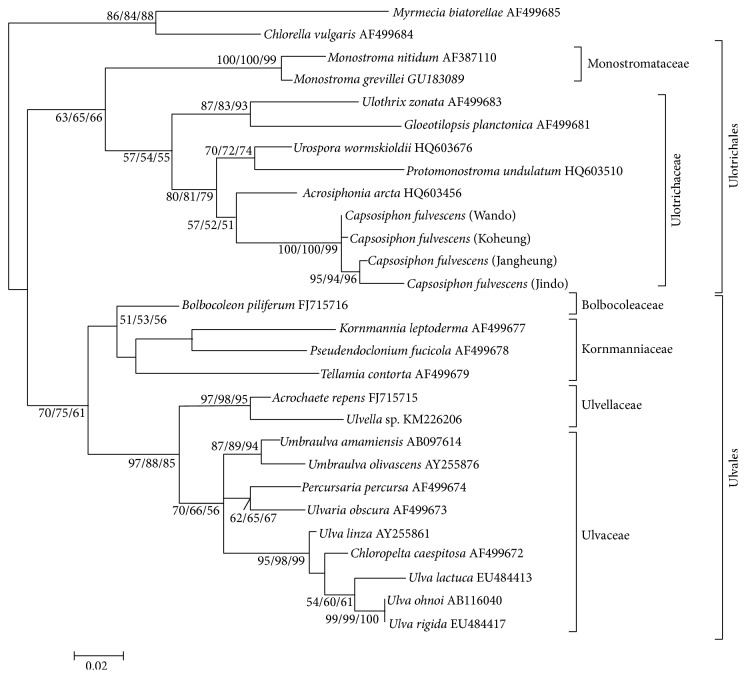
Maximum likelihood tree (−ln⁡*L* = 3240.16) of* rbc*L gene sequences of 26 species of Ulvophyceae. Numbers on branches represent maximum likelihood, neighbor joining, and maximum parsimony bootstrap support analysis, respectively (ML/NJ/MP). Scale bar: 0.02 (maximum composite likelihood).

*C. fulvescens rbc*L sequence was aligned with 25 previously published* rbc*L sequences which represent Ulvales and Ulotrichales in class Ulvophyceae (Figure 1).

Sequences of* Myrmecia biatorellae* and* Chlorella vulgaris* in class Trebouxiophyceae were used as outgroups (Figure 1). For phylogenetic analyses, 3 different methods were applied: neighbor joining (NJ), maximum parsimony (MP), and maximum likelihood (ML). To construct the NJ tree, 1,253 nucleotide positions were included. Heuristic searches under the MP criterion with 339 parsimony informative characters recovered the 3 most parsimonious trees (tree length [*L*]: 545, consistency index [CI]: 0.454, and retention index [RI]: 0.645). The ML tree inferred with the HKY DNA substitution model also recovered a tree with the 3240.16 −ln⁡*L* score. For clarity, only bootstrap numbers over 50% majority-rule consensus trees of MP and NP were shown on nodes in the ML tree. Within Ulotrichales, the clade comprising* Capsosiphon fulvescens* and* Protomonostroma undulatum* was strongly supported as sister to the clade comprised of* Pseudothrix borealis* and the* Urospora* accessions.

### 3.2. Constitution of 18S rDNA Phylogenetic Tree

**Figure 2:**
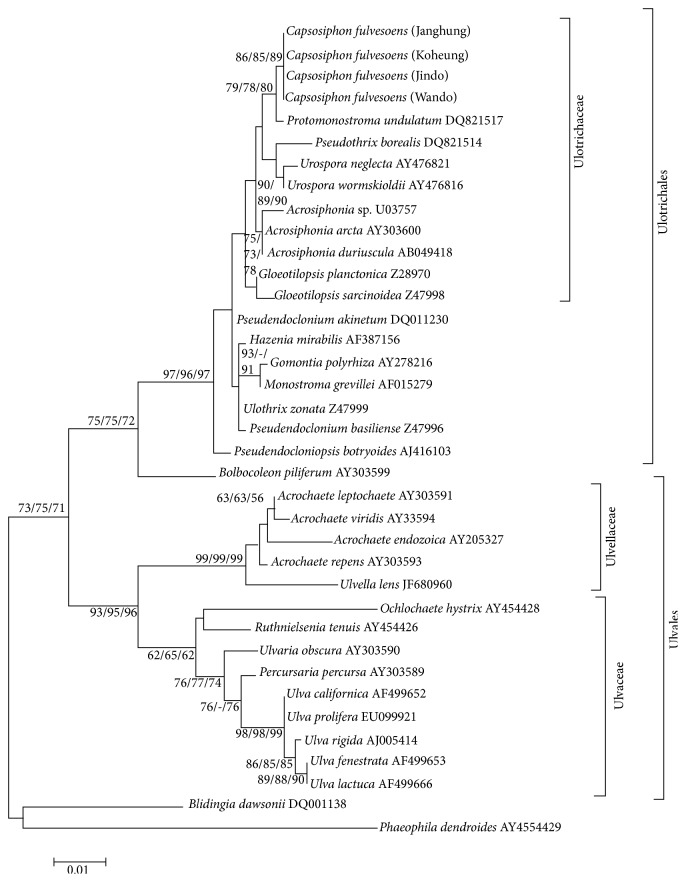
Maximum likelihood tree (−ln⁡*L* = 2022.00) of 18S rDNA gene sequences of 34 species of Ulvophyceae. Numbers on branches represent maximum likelihood, neighbor joining, and maximum parsimony bootstrap support analysis, respectively (ML/NJ/MP). Scale bar: 0.01 (maximum composite likelihood).

The* C. fulvescens* 18S rDNA sequences were aligned with 33 previously published green algal sequences (Figure 2) at 1,439 nucleotide positions after removing all gaps.

Of the 33 sequences, 13 sequences represented Ulotrichaceae, Gomontiaceae, Gayraliaceae, and Monostromataceae in Ultrichales and the remaining sequences represented Ulvellaceae, Kornmanniaceae, Bolbocoleaceae, Phaeophilaceae, and Ulvaceae in Ulvales. In the 18S rDNA analysis,* Capsosiphon fulvescens* occurred within a clade containing* Acrosiphonia*,* Urospora*,* Pseudothrix*, and* Protomonostroma*. It is closely related to* Protomonostroma* with strong support. HKY DNA substitution model was used to construct the ML tree with the score −ln⁡*L* = 2022.00.

## 4. Discussion

After Hayden and Waaland [13] reported that Ulvales and Ulotrichales sensu Floyd and O'Kelly [20] are monophyletic sister orders, the systematics in Ulvales has been well supported by molecular marker as 18S rDNA [21, 22] and shows 3 main families: Ulvaceae, Kornmanniaceae, and Ulvellaceae. However, classification and phylogenetic relationships in Ulotrichales are complicated. Despite several studies of the beneficial value of* C. fulvescens* to health, the position of* C. fulvescens* in the order Ulotrichales is still unclear. Phylogenetic trees were constructed on the basis of* rbc*L, 18S rDNA, and combined sequences. This information has been further used for phylogenetic analysis and classification of Ulvophyceae and other related taxa.

Phylogenetic trees based on* rbc*L and 18S rDNA sequences exhibit topological differences that are due to the different rates at which these genes evolve. To make any statement from our phylogenetic trees, our study only selected the tree topology supported by >50% in at least 2 different phylogenetic trees. The* rbc*L tree (Figure 1) recovered Ulvales as a monophyletic group, which was consistent with the previous studies and showed that* C. fulvescens* was weakly supported in Ulotrichales because all phylogenetic trees have showed a corresponding topology with at least 50% of bootstrap value.

The 18S rDNA phylogenetic tree (Figure 2) provided more information about the phylogenetic systematics of Ulotrichales and the position of* C. fulvescens*.* Capsosiphon fulvescens* appeared on its own branch of the Ulotrichales in the NJ and ML analysis; in the MP analysis, it appeared on its own branch at the base of the Ulotrichaceae. In all analyses, it clearly belonged to the Ulotrichales since the subtending branch, separating the Ulotrichales from members of the Ulvales, had 70% bootstrap support.

Our phylogenetic tree shows that Ulotrichales appears to be paraphyletic. Two clearly different lineages in Ulotrichaceae (*Acrosiphonia* and* Urospora*) were also successfully recovered with >80% of bootstrap numbers in all 3 phylogenetic methods (ML/MP/NJ = 81/83/85), which was also consistent with the SSU rDNA tree [14, 21]. The key result here is that the tree topology in Ulotrichaceae shows 4 distinct lineages:* Gloeotilopsis*,* Acrosiphonia*,* Capsosiphon*, and* Urospora* +* Protomonostroma* (Figure 2). This tree topology [*Acrosiphonia*, and (*Capsosiphon*,* Urospora* +* Protomonostroma*)] was supported by >50% of bootstraps in all 3 different phylogenetic trees, suggesting that* Capsosiphon* is more closely related to* Urospora* +* Protomonostroma*. In both MP and NJ analyses with the 18S rDNA data set, 3 well-supported groups were recovered: (1) Gomontiaceae, (2) Monostromataceae, and (3) Ulotrichaceae. The relationship between Ulotrichaceae and other genera is not well supported in either NJ or MP tree. In NJ tree, the proximal outgroup Ulotrichaceae and Gomontiaceae + Monostromataceae are similar to the phylogram including 5 representatives of Kornmanniaceae [22].

The present study showed that both phylogenetic analysis based on 18S rDNA and* rbc*L sequences analysis resolved the phylogenetic position of* C. fulvescens* in Ulotrichales (Figures 1 and 2). All of 4* C. fulvescens* were clustered with* Urospora wormskioldii* and* Acrosiphonia arcta* in the family Ulotrichaceae, Ulotrichales, with high bootstrap number in all phylogenetic trees (Figure 2), suggesting that* Capsosiphon* is a genus in the order Ulotrichales, although additional information is needed to support this hypothesis. We will further study the vegetative morphology of this genus to compare it with that of known genera in the Ulotrichales.

## Supplementary Material

Supplementary data 1. *rbc*L and 18S rDNA sequences of *Capsosiphon fulvescens* collected from Jindo, Koheung, Wando and Jangheung, South Korea.

## Figures and Tables

**Table 1:** PCR and sequencing primers used in the present study.

Primer	Sequence	Target	Direction
RH1^a^	5′-ATGTCACCACAAACAGAAACTAAAGC-3′	*rbc*L	Forward
Rbc571^c^	5′-TGTTTACGAGGTGGTCTTGA-3′	*rbc*L	Forward
*rbc*L-LongF	5′-ATTCCAAGGTCCTCCACACGG-3′	*rbc*L	Forward
Rbc590^c^	5′-TCAAGACCACCTCGTAAACA-3′	*rbc*L	Reverse
1385r^a^	5′-AATTCAAATTTAATTTCTTTCC-3′	*rbc*L	Reverse
*rbc*L-LongR	5′-GCAGTCAATTCAGGACTCCATTTACAAGC-3′	*rbc*L	Reverse

AB1^b^	5′-GGAGGATTAGGGTCCGATTCC-3′	18S	Forward
18S-F1	5′-TTCATTGATCAAGAACGAAAGYYGGG-3′	18S	Forward
18S-5′END-F2	5′-GTCATATGCTTGTCTCAAAGATTAAGCC-3′	18S	Forward
18S-3′END-F3	5′-GACGATTAGATACCGTCGTAGTCTCAAC-3′	18S	Forward
18S-R1	5′-GCAGGGACGTAATCAACGCGA-3′	18S	Reverse
18S-5′END-R1	5′-CCTTGTTACGACTTCTCCTTCCTCTAA-3′	18S	Reverse

^a^Manhart 1994 [16].

^b^van Oppen 1995 [17].

^c^Hayden and Waaland 2002 [13].
