# Supplementary material for: Reconstructing the Phylogeny of Capsosiphon fulvescens (Ulotrichales, Chlorophyta) from Korea Based on rbcL and 18S rDNA Sequences
Source: Biomed Res Int. 2016 Apr 17;2016:1462916. doi: 10.1155/2016/1462916 (PMC4852126; doi:10.1155/2016/1462916)
Supplement: Supplementary file 1 — Supplementary data 1. rbcL and 18S rDNA sequences of Capsosiphon fulvescens collected from Jindo, Koheung, Wando and Jangheung, South Korea. [file 1462916.f1.pdf]

Supplementary data 1. *rbc*L and 18S rDNA sequences.

***rbc*L Sequences**

Capsosiphon fulvescens (Jindo) (994 bp)

agcaggaactggcttcaaagctggtgttaaagactaccgtttaacttactacacacctgattatcaagtaaaagatactgatattttagctgcattccgtatgtc
acctcaaccaggtgtacctgcagaagaagctggtgctgcagtagctgctgaatcatcaaccggaacttggactacagtatggactgatggtttaacatcttt
agaccgttataaaggacgttgttatgacatcgaacctgtagctggtgaagataaccaatatattgcttatattgcttacccattagacttatttgaagaaggttct
gtaacaaacctatttacttcaattgtaggtaacgtttttggatttaaagctttacgtgctttacgtttagaagatttacgtgttccaccagcatacactaaaacattc
caaggtcctccacacgggattcaggtagaacgtgacaaactaaacaaatatggtcgtggtctattaggttgtacaattaaaccaaaattaggttatacttcatt
agcaatttactgtcgtaacaatgatcttttattacacattcaccgtgcaatgcacgctgtaattgaccgtcaacgtaaccatggtattcacttccgtgttctagct
aaaattctacgtatgtcaggggggtgaccacttacactctggtactgttgtaggtaaactagaaggtgaacgtgaaattactttagatttcgtagatctaatgcgt
gatgactatattgaaaaagaccgtagtcgtggtatttacttcacacaagactgggtttcacttcctggtactatgccagtagcatcggggggtatccacgtat
ggcacatgccagcactagtggaaatctttggggaggaggcttgtttacaattcggggggggtactttaggtcacccagggggtaatgctccaggggcag
ctgctaaccgggtagctttagaagcagtgtactcaagctggtaacgaaggtcgtga

Capsosiphon fulvescens (Koheung) (1,174 bp)

agcaggaactggcttcaaagctggtgttaaagactaccgtttaacttactacacacctgattatcaagtaaaagatactgatattttagctgcattccgtatgtc
acctcaaccaggtgtacctgcagaagaagctggtgctgcagtagctgctgaatcatcaaccggaacttggactacagtatggactgatggtttaacatcttt
agaccgttataaaggacgttgttatgacatcgaacctgtagctggtgaagataaccaatatattgcttatattgcttacccattagacttatttgaagaaggttct
gtaacaaacctatttacttcaattgtaggtaacgtttttggatttaaagctttacgtgctttacgtttagaagatttacgtgttccaccagcatacactaaaacattc
caaggtcctccacacgggattcaggtagaacgtgacaaactaaacaaatatggtcgtggtctattaggttgtacaattaaaccaaaattaggtttatcagcta
aaaactacgggcgtgccgtttacaaatgtttctaccaaagatgatgaaaacctaaactccccaccctttatgccttggcgtgaccgtttcttattcctagcaga
agccatttaccaatcctcagctgaaactggtgaagttaaaggtcactacttaaatgcaacagctggtacttgtgaagaaatgatgttccgtgcagaatttgcg
aaagatttaggtgttccaattattatgcatgactaccttacaggtggttttactgcgaatacttcattagcaatttactgtcgtaacaatggtcttttattacacattc
accgtgcaatgcacgctgtaattgaccgtcaacgtaaccatggtattcacttccgtgttctagctaaaattctacgtatgtcaggggggtgaccacttacactct
ggtactgttgtaggtaaactagaaggtgaacgtgaaattactttaggtttcgtagatctaatgcgtgatgactatattgaaaaagaccgtagtcgtggtatttac
ttcacacaagactgggtttcacttcctggtactatgccagtagcatctggtggtatccacgtatggcacatgccagcactagttgaaatctttggtgatgatgc
ttgtttaccattcggtggtgg

Capsosiphon fulvescens (Wando) (1,284 bp)

agcaggaactggcttcaaagctggtgtaaagattactatttaacttattacacgcctgattatcaagtaaaagatactgatattttagctgcgttccgtatgactc
ctcaaccaggagtacctgcagaagaagctggtgctgctgttgctgctgaatcatcaaccggaacttggactactgtatggactgatggtttaacatctttaga
tcgttataaaggacgttgttacgacattgaaccattaggagaagacgaccaatatattgcttatattgcttacccattagacttatttgaagaaggttctgtaaca
aacctatttacttcaattgtaggtaacgtttttggatttaaagctttacgtgctttacgtttagaagatttacgtgttccaccagcatacactaaaacattccaaggt
cctccacacgggattcaggtagaacgtgacaaactaaacaaatatggtcgtggtctattaggttgtacaattaaaccaaaattaggttaccaaaattcggttt
atcagctaaaaactacgggcgtgccgtttacgaacgtttacgcggtggtttagactttacaaaagatgatgaaaacgtaaactcacaaccatttatgcgttgg
cgtgaccgtttcttattcgtagcagaagcaatttacaaatctcaagctgaaactggtgaagttaaaggtcactacttaaatgcaacagctggtacttgtgaag
aaatgatgttccgtgcagaatttgcgaaagatttaggtgttccaattattatgcatgactacattacaggtggttttactgcgaatacttcattagcaatttactgt
cgtaacaatggtcttttattacacattcaccgtgcaatgcacgctgtaattgaccgtcaacgtaaccatggtattcacttccgtgttctagctaaaattctacgta
tgtcaggggggtgaccacttacactctggtactgttgtaggtaaactagaaggtgaacgtgaaattactttaggtttcgtagatctaatgcgtgatgactatattg
aaaaagaccgtagtcgtggtatttacttcacacaagactgggtttcacttcctggtactatgccagtagcatctggtggtatccacgtatggcacatgccagc
actagttgaaatctttggtgatgatgcttgtttacaattcggtggtggtactttaggtcacccatggggtaatgctccaggtgcagctgctaaccgtgtagcttt
agaagcatgtactcaagctcgtaacg

Capsosiphon fulvescens (Jangheung) (1,258 bp)

agcaggaactggcttcaaagctggtgttaaagactaccgtttaacttactacacacctgattatcaagtaaaagatactgatattttagctgcattccgtatgtc
acctcaaccaggtgtacctgcagaagaagctggtgctgcagtagctgctgaatcatcaaccggaacttggactacagtatggactgatggtttaacatcttt
agaccgttataaaggacgttgttatgacatcgaacctgtagctggtgaagataaccaatatattgcttatattgcttacccattagacttatttgaagaaggttct
gtaacaaacctatttacttcaattgtaggtaacgtttttggatttaaagctttacgtgctttacgtttagaagatttacgtgttccaccagcatacactaaaacattc
caaggtcctccacacgggattcaggtagaacgtgacaaactaaacaaatatggtcgtggtctattaggttgtacaattaaaccaaaattaggtttatcagcta
aaaactacgggcgtgccgtttacaaatgtttctttacaaaagatgatgaaaacgtaaactcacaaccatttatgcgtgggcgtgaccgtttcttattcgtagca
gaagcaatttacaaatctcaagctgaaactggtgaagttaaaggtcactacttaaatgcaacagctggtacttgtgaagaaatgatgttccgtgcagaatttg
cgaaagatttaggtgttccaattattatgcatgactacattacaggtggttttactgcgaatacttcattagcaatttactgtcgtaacaatggtcttttattacaca
ttcaccgtgcaatgcacgctgtaattgaccgtcaacgtaaccatggtattcacttccgtgttctagctaaaattctacgtatgtcaggggggtgaccacttacac
tctggtactgttgtaggtaaactagaaggtgaacgtgaaattactttaggtttcgtagatctaatgcgtgatgactatattgaaaaagaccgtagtcggggtatt
tacttcacacaagactgggtttcacttcctggtactatgccagtagcatctggtggtatccacgtatggcacatgccagcactagtggaaatctttggggatg
aggcttgtttacaattcggtggtggtactttaggtcacccatggggtaatgctccaggtgcagctgctaaccgtgtagctttagaagcaggtactcaagctg
gtaac

18S rDNA Sequences

Capsosiphon fulvescens (Jindo) (1,227 bp)

gcaggcgcgcaattacccaatcctgacacagggaggtagtgacaataaatatcgatactgggccttcgggtccggtaattggaatgagtacaatctaaat
cccttaacgaggatccattggagggcaagtctggtgccagcagccgcggtaattccagctccaatagcgtatatttaagttgttgcagttaaaaagctcgta
gttggatttcgggtgggtaccgccggtctccctttgggtatgtactggcgtggcccgccttgctgccggggacgatctcctgggcttaactgtccgggaat
cggagtcggcgatgttactttgagtaaattagagtgttcaaagcaagcctacgctctgaatataatagcatgggataacacgacaggactctggcctatcgt
gttggtctgtaggaccggagtaatgattaagagggacagtcgggggcattcgtattccattgtcagaggtgaaattcttggatttatggaagacgaacatct
gcgaaagcatttgtcaaggatgttttcattgatcaagaacgaaagttgggggctcgaagacgattagataccgtcgtagtctcaaccataaacgatgccga
ctggggattggcggatgtttgtttgatgactccgccagcaccttatgagaGggttccgggggagtatggtcgcaaggctgaaacttaaaggaattgacg
gaagggcaccaccaggcgtggagcctgcggcttaatttgactcaacacgggaaaacttaccaggtccagacataggaaggattgacagattgatagctc
tttcttgattctgtgggtggtggtgcatggccgttcttagttggtgggttgccttgtcaggttgattccggtaacgaacgagacctcagcctgctaaatagtga
cgattgctttggcagttggcccgcttcttagagggactgttggcgtctagccaatggaagtatgaggcaataacaggtctgtgatgcccttagatgttctgg
gccgcacgcgcgctacactgatacgttcaacaagttcctaggccgaaaggtccgggtaatctttgaaaccgtatcgtgatggggatagaacattgcaatta
ttgttcttcaacgaggaatgcctagtaagcgtgagtcatcatctcgcgttgattacgtccctgccctttgtacacaccgcccgtcgctcctaccgattgaacgt
gctggtgaa

Capsosiphon fulvescens (Koheung) (1,291 bp)

gcaggcgcgcaattacccaatcctgacacagggaggtagtgacaataaatatccatactgggccttcgggtccggtaattggaatgagtacaatctaaat
cccttaacgaggatccattggagggcaagtctggtgccagcagccgcggtaattccagctccaatagcgtatatttaagttgttgcagttaaaaagctcgta
gttggatttcgggtgggtaccgccggtctccctttgggtatgtactggcgtggcccgccttgctgccggggacgatctcctgggcttaactgtccgggaat
cggagtcggcgatgttactttgagtaaattagagtgttcaaagcaagcctacgctctgaatataatagcatgggataacacgacaggactctggcctatcgt
gttggtctgtaggaccggagtaatgattaagagggacagtcgggggcattcgtattccattgtcagaggtgaaattcttggatttatggaagacgaacatct
gcgaaagcatttgtcaaggatgttttcattgatcaagaacgaaagttgggggctcgaagacgattagataccgtcgtagtctcaaccataaacgatgccga
ctagggattggcggatgtttgtttgatgactccgccagcaccttatgagagggttccgggggagtatggtcgcaaggctgaaacttaaaggaattgacg
gaagggcaccaccaggcgtggagcctgcggcttaatttgactcaacacgggaaaacttaccaggtccagacataggaaggattgacagattgatagctc
tttcttgattctgtgggtggtggtgcatggccgttcttagttggtgggttgccttgtcaggttgattccggtaacgaacgagacctcagcctgctaaatagtga
cgattgctttggcagttggcccgcttcttagagggactgttggcgtctagccaatggaagtatgaggcaataacaggtctgtgatgcccttagatgttctgg
gccgcacgcgcgctacactgatacgttcaacaagttcctaggccgaaaggtccgggtaatctttgaaaccgtatcgtgatggggatagaacattgcaatta
ttgttcttcaacgaggaatgcctagtaagcgtgagtcatcatctcgcgttgattacgtccctgccctttgtacacaccgcccgtcgctcctaccgattgaacgt

gctggtgaagagttcggattggagtcttggctaggtttcctagccctgattccgagaagttctttaaaccctc

Capsosiphon fulvescens (Wando) (1,284 bp)

gcaggcgcgcaaattacccaatcctgacacagggaggtagtgacaataaatatcaatacagggccttcgggtccggtaattggaatgagtacaatctaaa
tcccttaacgaggatccattggagggcaagtctggtgccagcagccgcggtaattccagctccaatagcgtatatttaagttgttgcagttaaaaagctcgt
agttggatttcgggtgggtaccgccggtctccctttgggtatgtactggcgtggcccgccttgctgccggggacgatctcctgggcttaactgtccgggaa
tcggagtcggcgatgttactttgagtaaattagagtgttcaaagcaagcctacgctctgaatataatagcatgggataacacgacaggactctggcctatcg
tgttggtctgtaggaccggagtaatgattaagagggacagtcgggggcattcgtattccattgtcagaggtgaaattcttggatttatggaagacgaacatct
gcgaaagcatttgtcaaggatgttttcattgatcaagaacgaaagttgggggctcgaagacgattagataccgtcgtagtctcaaccataaacgatgccga
ctagggattggcggatgtttgtttgatgactccgccagcaccttatgagaaatcaaaagttttgtccTgactccgccagcaccttatgagaaatcaaagttttt
gggttccgggggagtatggtcgcaaggctgaaacttaaaggaattgacggaagggcaccaccaggcgtggagcctgcggcttaatttgactcaacac
gggaaaacttaccaggtccagacataggaaggattgacagattgatagctctttcttgattctgtgggtggtggtgcatggccgttcttagttggtgggttgc
cttgtcaggttgattccggtaacgaacgagacctcagcctgctaaatagtgacgattgctttggcagttggcccgcttcttagagggactgttggcgtctag
ccaatggaagtatgaggcaataacaggtctgtgatgcccttagatgttctgggccgcacgcgcgctacactgatacgttcaacaagttcctaggccgaaa
ggtccgggtaatctttgaaaccgtatcgtgatggggatagaacattgcaattattgttcttcaacgaggaatgcctagtaagcgtgagtcatcatctcgcgtt
gattacgtccctgccctttgtacacaccgcccgtcgctcctaccgattgaacgtgctggtgaag

Capsosiphon fulvescens (Jangheung) (1,240 bp)

gcaggcgcgcaaattacccaatcctgacacagggaggtagtgacaataaatatcattactgggccttcgggtccggtaattggaatgagtacattctaaat
cccttaacgaggatccattggagggcaagtctggtgccagcagccgcggtaattccagctccaatagcgtatatttaagttgttgcagttaaaaagctcgta
gttggatttcgggtgggtaccgccggtctccctttgggtatgtactggcgtggcccgccttgctgccggggacgatctcctgggcttaactgtccgggaat
cggagtcggcgatgttactttgagtaaattagagtgttcaaagcaagcctacgctctgaatataatagcatgggataacacgacaggactctggcctatcgt
gttggtctgtaggaccggagtaatgattaagagggacagtcgggggcattcgtattccattgtcagaggtgaaattcttggatttatggaagacgaacatct
gcgaaagcatttgtcaaggatgttttcattgatcaagaacgaaagttgggggctcgaagacgattagataccgtcgtagtctcaaccataaacgatgccga
ctagggattggcggatgtttgtttgatgactccgccagcaccttgacggaagggcaccaccaggcgtggagcctgcggcttaatttgactcaacacggga
aaacttaccaggtccagacataggaaggattgacagattgatagctctttcttgattctgtgggtggtggtgcatggccgttcttagttggtgggttgccttgt
caggttgattccggtaacgaacgagacctcagcctgctaaatagtgacgattgctttggcagttggcccgcttcttagagggactgttggcgtctagccaat
ggaagtatgaggcaataacaggtctgtgatgcccttagatgttctgggccgcacgcgcgctacactgatacgttcaacaagttcctaggccgaaaggtcc
gggtaatctttgaaaccgtatcgtgatggggatagaacattgcaattattgttcttcaacgaggaatgcctagtaagcgtgagtcatcatctcgcgttgattac
gtccctgccctttgtacacaccgcccgtcgctcctaccgattgaacgtgctggtgaagagttcggattggagtcttggctaggtttcctagccctgattccga

gaagttctttaaaccctc
